# The Hyperglycemia and Hyperketonemia Impaired Bone Microstructures: A Pilot Study in Rats

**DOI:** 10.3389/fendo.2020.590575

**Published:** 2020-10-22

**Authors:** Qi Liu, Zhou Yang, Chuhai Xie, Long Ling, Hailan Hu, Yanming Cao, Yan Huang, Qingan Zhu, Yue Hua

**Affiliations:** ^1^ Department of Orthopaedic Surgery, The Second Affiliated Hospital of Guangzhou Medical University, Guangzhou, China; ^2^ Department of Orthopaedic Surgery, Southern University of Science and Technology Hospital, Shenzhen, China; ^3^ Division of Spine Surgery, Department of Orthopedics, Nanfang Hospital, Southern Medical University, Guangzhou, China; ^4^ School of Traditional Chinese Medicine, Southern Medical University, Guangzhou, China

**Keywords:** diabetes mellitus, ketogenic diet, cancellous bone, cortical bone, bone turnover

## Abstract

Though diabetes mellitus (DM) is one of the known causes of osteoporosis, it is also realized that ketogenic diet (KD), an effective regimen for epilepsy, impairs bone microstructures. However, the similarities and differences of effects between these two factors are still unknown. The purpose of this study is to identify different effects between hyperglycemia and hyperketonemia, which are manifestations of DM and KD, on bone in rats. Thirty male Sprague-Dawley rats were randomly divided into three groups: the sham, DM, and KD groups. Hyperglycemia was achieved by intravenous injection of streptozotocin in DM group, while hyperketonemia was induced by application of ketogenic diet (carbohydrates-to-fat as 1:3) in KD group. The body weight, blood ketone body, and blood glucose were recorded, and the bone turnover markers, bone length, bone microstructures, bone biomechanics and histomorphology were measured after 12 weeks intervention. Compared with the control and KD groups, a significant body weight loss was found in the DM group, and the bone lengths of tibia and femur of the group were shortened. The blood glucose and blood ketone were noticeably increased in the DM and KD rats, respectively. Microstructures and properties of cancellous bone were significantly deteriorated in both the DM and KD groups compared with the sham group, as the bone volumes were decreased and the bone trabecula structures were disturbed. Meanwhile, the thickness and strength of cortical bone was reduced more in the DM group than those in the sham and KD groups. The HE staining showed that bone trabecula was significantly decreased in both the DM and KD groups, and more adipose tissue was observed in the KD rats. The activity of osteoblasts was decreased more in both the KD and DM groups than that in the sham group, while the activity of osteoclasts of the two groups was remarkably increased. The present study indicates that both hyperglycemia and hyperketonemia have adverse effects on bone. Therefore, it is worth paying more attention to the bone status of patients with hyperglycemia and hyperketonemia in clinic.

## Introduction

Metabolic syndrome and osteoporosis are medical conditions that must not be overlooked, since both may lead to serious problems in the aged (1). Metabolic syndrome is characterized by abdominal obesity, impaired glucose tolerance, hypertension and dyslipidemia (2). The accumulating studies reported that metabolic disturbance might be a risk factor for osteoporosis. Increased insulin resistance promotes osteolysis of inflammatory cytokines in patients with metabolic syndrome, triggering osteoporosis (3). Moreover, higher serum lipid level resulting from metabolic syndrome is considered to play a critical factor in pathogenesis of osteoporosis (4).

Diabetes mellitus is a metabolic disease which is characterized as hyperglycemia resulted from defects in insulin action and/or insulin secretion (5). It has negative effects on skeletal disorders, including osteopenia or osteoporosis (6). It is known that type 1 diabetes mellitus (T1DM) compromises bone microstructures by suppressing growth potential and worsening insulin resistance and glycemic control (7). By carrying out an MRI-based assessment, Naiemh et al. (8) found that young women with T1DM were more likely to suffer from trabecular bone volume and trabecular numbers reduction. Shortened femur length, reduced growth plate thickness, compromised cancellous and cortical bone, and decreased collagen II expression were observed in streptozotocin-induced diabetic animal models (9, 10).

Ketogenic diet (KD) is a well-established therapeutic intervention for patients with refractory epilepsy (11). By maintaining a high-fat and low-carbohydrate diet with restricted protein intake, systemic ketosis raises seizure threshold for intractable epilepsy treatment (12). However, it was firstly reported by Hahn that KD had deleterious effects on bone mass in children who maintained this regimen in long term (13). Our previous studies also confirmed that KD compromised bone micro-structures and reduced bone biomechanics in rodents (14–18). However, the different effects of streptozotocin-induced hyperglycemia and KD-induced hyperketonemia on bone micro-structures and biomechanics have not been elucidated.

The purpose of this study is to investigate the effects of hyperglycemia and hyperketonemia on appendicular bone. Micro-CT scan and biomechanical test were performed to evaluate the bone mass and biomechanical properties, and both the serological and histological bone turnover markers were observed to evaluate the activities of osteoblasts and osteoclasts after intervention.

## Materials and Methods

### Animals

A total of thirty Sprague Dawley male rats, purchased at 12 weeks of age from the Experimental Animal Center of Southern Medical University, were randomly divided into three groups: the sham group, the diabetes mellitus (DM) group, and the ketogenic diet (KD) group. The rats in the DM group were injected intravenously with streptozotocin (STZ; Fisher Scientific) at the dose of 50 mg/kg (10), while the rats in the KD group were fed with the ketogenic diet (Jielikang Inc., Shenzhen, China), which containing a ratio of fat to carbohydrate and protein of 3:1. All rats were kept in a wire hanging cage with a 12-h light–dark cycle, and a constant temperature of 25°C and humidity of 48%. And they had free access to food and water during the entire experiment.

### Specimens Collection

All the rats were anesthetized by 1% pentobarbitone sodium after 12-weeks interventions. The blood samples were collected with a 5 ml syringe from abdominal vein, and then centrifuged under 3000rpm for 20 min to obtain the serum samples. The left tibiae and left femora were fixed in 4% p-formaldehyde for 48 h before analyzing the bone microstructures, histology and immunohistochemistry, and the right tibiae and right femora were frozen in -20°C until biomechanical properties analysis.

### Measurement of Body Weight, Length of Tibia and Femur, Blood Ketone Body, and Blood Glucose

The rats were weighed at 12 weeks using a CS 200 balance (Ohaus, Pine Brook, NJ, USA). The full lengths of the tibia and femur were measured with a vernier caliper. The blood ketone and glucose levels were measured by cutting tail veins. Yicheng Blood Ketone Meter T-1 (Sentest Inc., China) and Medisense Precision Xtra monitor (Abbott Laboratories, Canada) were used to determine the blood ketone concentrations, while the blood glucose levels were tested with monitor JPS-5 (Leapon Inc., China).

### Analysis of Bone Turnover Biomarkers in the Serum

The collected serum samples were used to test the bone turnover biomarkers. The serological calcium and phosphorus concentrations, as well as the concentrations of the specific markers of bone turnover biomarkers, including bone-specific alkaline phosphatase (ALP), tartrate-resistant acid phosphatase (TRAP), insulin-like growth factor 1 (IGF-1), N-terminal propeptide of type I procollagen (P1NP), and C-telopeptide fragments of collagen type I a1 chains (β-CTX), were measured by available assay kit (Beckman Coulter, Suzhou, China) according to the manufacturer’s protocols.

### Micro-CT Scan

Each specimen was washed with tap water for 2 h and kept straight in the tube for micro-CT scanning after fixation. Refer to the guidelines for assessment of the bone microstructures in rodents using micro-CT (19), the trabecular microstructures of left tibiae were analyzed using a micro-CT system (μCT80, SCANCO MEDICAL, Switzerland) at resolutions of 12 μm with a tube voltage of 50 kV and a tube current of 0.1 mA. The region of interest (ROI) was defined as 180 slices approximately 2.0 mm from the growth plate of the proximal tibia. Bone morphometric parameters, included the tissue mineral density (TMD), the connection density (Conn.D), the bone volume/tissue volume (BV/TV), the trabecular number (Tb.N), the trabecular thickness (Tb.Th) and the trabecular separation (Tb.Sp), were obtained *via* analysis of the ROI. The 180 slices of left femur mid-diaphysis were selected to analyze the parameters of cortical bone, including the total cross-sectional area inside the periosteal envelope (Tarea), the bone area (Barea) and the thickness (Ct.Th).

The micro-finite element analysis (micro-FEA) of compressive test (SCANCO Medical AG, Version 1.13) was used to assess the biomechanical characteristics in cancellous bone based on the micro-structures from micro-CT images. The simulations were done within the framework of linear elasticity, and the detailed operation was shown in our previous study (17). The simulation yielded the compressive stiffness and failure load of cancellous bones.

### Three-Point Bending Test

The three-point bending test was performed to evaluate the biomechanical properties of cortical bone. After thawing 1 h at room temperature, the proximal tibiae were placed on a base consisting of an aluminum block with one rounded edge-free notches on top and the mid-shaft of femora were placed on two supports separated by a distance of 20 mm for biomechanical test. A compressive force was applied at a constant speed of 2 mm/min by the material testing machine (Instron ElectroPuls, E1000, USA). The maximum load (Max.L), stiﬀness, and energy absorption were obtained based on the load-deformation curve after the specimen was broken.

### Histological and Immunohistochemical Staining

The proximal tibiae were embedded into olefin after micro-CT scan, and then decalcification in 10% EDTA for 4–5 weeks. All samples were stained with the hematoxylin–eosin and immunohistochemical (IHC) staining according to standard conditions. The hematoxylin–eosin staining was performed to observe the histomorphology of trabecula, while the osteocalcin (OCN) staining (Abcam Cambridge, UK) and the tartrate-resistant acid phosphatase (TRAP) staining (Sigma–Aldrich, St. Louis, USA) were performed to evaluate the osteoblasts cell activity and the presence of osteoclasts in the trabecular bone. The results of IHC staining were evaluated by cell number counting and computerized optical density (OD) measurements. Cells per bone surface (B.S) were used to calculate the number of positive cells, and integrated optical density per area of positive cells (IOD/area, mean density) was used to semi-quantify the staining intensity by detecting in 10 diﬀerent images taken at 400× magnification every slide with Image Pro Plus 6.0 software (Media Cybernetics, MD, USA).

### Statistical Analysis

All data were analyzed by SPSS 20.0 software and showed as mean ± SD. The diﬀerences in the body weight, the blood ketone and glucose levels, the serological biomarkers, the micro-CT parameters, the biomechanical results, and the histological results were analyzed by one-way ANOVA with least-significant diﬀerence (LSD) *post hoc* test among groups. P<0.05 was considered statistically significant.

## Results

### Changes of Body Weight, Blood Ketone and Blood Glucose Concentrations, and Bone Lengths

The detailed data of body weight and the levels of blood ketone and glucose are shown in **Table 1**. The body weight of the DM group was significantly decreased compared with the sham and KD groups (P<0.05), while no significant difference was shown between the sham group and the KD group (P>0.05). The blood ketone of the KD group was noticeably higher than that of the sham group or the DM group (the blood ketone concentrations were 0.40 ± 0.14mmol/L, 0.55 ± 0.21mmol/L and 1.13 ± 0.12mmol/L in the sham, DM and KD groups, respectively). The DM rats exhibited higher blood glucose levels than those in the sham and KD groups (the blood glucose concentrations were 7.10 ± 1.15 mmol/L, 6.1 ± 0.47 mmol/L and >33.33 mmol/L in the sham, KD and DM groups, respectively).

**Table 1:** The body weight, blood glucose, and blood ketone among groups.

Groups	Body weight(g)	Blood Ketone(mmol/L)	Blood Glucose(mmol/L)
Sham	508.33 ± 15.41	0.40 ± 0.14	7.10 ± 1.15
DM	290.12 ± 50.15*	0.55 ± 0.21	>33.33*
KD	507.33 ± 22.23	1.13 ± 0.12*	6.1 ± 0.47

Data are represented as means ± SD. “*” means P < 0.05 compared to Sham group.

**Figure 1:**
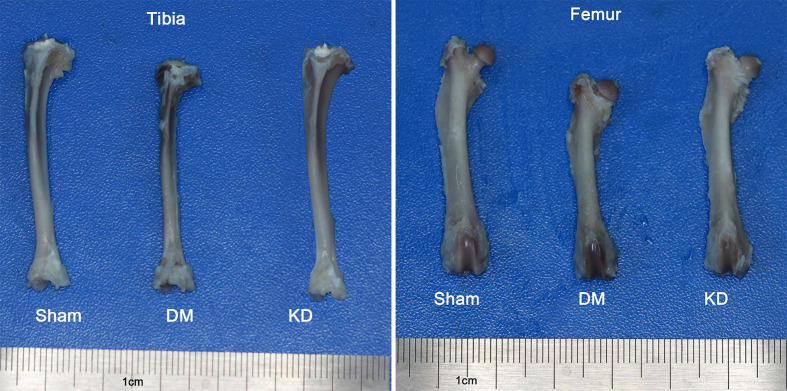
The general picture of bone length of tibia and femur in the sham, diabetes mellitus (DM) and ketogenic diet (KD) groups.

The bone length showed a trend similar to the body weight. The tibia and femur lengths of DM group were the shortest among all the groups (**Figure 1**). The lengths of tibia and femur of DM group were 0.47mm and 0.52mm, respectively, shorter than those in the sham group (**Table 2**).

**Table 2:** The bone length of tibia and femur among groups.

Groups	Tibia (mm)	Femur (mm)
Sham	4.52 ± 0.15	4.10 ± 0.12
DM	4.05 ± 0.09*	3.58 ± 0.11*
KD	4.36 ± 0.11	4.00 ± 0.08

Data are represented as means ± SD. “*” means P < 0.05 compared to Sham group.

### Analysis of Serum Calcium, Phosphorus, and Bone Turnover Biomarkers

The serum concentrations of calcium and phosphorus showed no significant difference among the groups, yet there were great differences among the bone turnover biomarkers (**Table 3**). The ALP level was significantly decreased in the KD group compared with the sham group (the ALP level was 0.78 fold in the DM group relative to the sham group, P<0.05). The concentrations of TRAP were increased remarkably in both DM and KD groups than that in the sham group (the TRAP levels were 113.0 μmmol/L, 206.3 μmmol/L and 192.3 μmmol/L in the sham, DM and KD groups, respectively).

**Table 3:** The serum parameters of calcium, phosphate, and bone turnover biomarkers among groups.

Parameters	Sham	DM	KD
Calcium (mol/L)	2.22 ± 0.09	2.21 ± 0.11	2.25 ± 0.08
Phosphorus (mol/L)	2.28 ± 0.32	2.24 ± 0.27	2.13 ± 0.20
ALP (μmol/L)	3.01 ± 0.34	2.65 ± 0.18*	2.25 ± 0.39*
TRAP (μmol/L)	113.02 ± 6.18	206.25 ± 41.21*	192.34 ± 19.67*
IGF-1 (ng/ml)	629.04 ± 66.53	230.84 ± 54.02*	339.51 ± 64.51*
P1NP (ng/ml)	12.83 ± 1.86	5.62 ± 0.90*	8.83 ± 1.19*
β-CTX (ng/ml)	16.02 ± 2.72	38.93 ± 10.20*	22.27 ± 2.95*^#^

Data are represented as means ± SD. “*” means P < 0.05 compared to Sham group, and “#” means P < 0.05 between DM and KD groups.

IGF-1, as a growth factor rich in bone tissue, stimulates proliferation of preosteoblastic cells and enhances differentiated functions of the osteoblast. The concentrations of IGF-1 were significantly decreased in the DM and KD rats compared with that in the sham rats (629.0 ng/ml, 230.8 ng/ml, and 339.5 ng/ml in the sham, DM and KD groups, respectively). P1NP and β-CTX are biomarkers of bone formation and resorption, which are recommended for clinical application. The concentrations of P1NP were decreased by 56.2% and 31.2% in the DM and KD groups compared with the sham group, while the β-CTX levels were increased by 143.1% and 39% in the DM and KD groups, respectively (**Table 3**). Moreover, the β-CTX concentration of the DM group was increased more significantly than that of the KD group (38.93 ng/ml and 22.27 ng/ml in the DM and KD groups, respectively).

### Changes of Bone Microstructures

**Figure 2:**
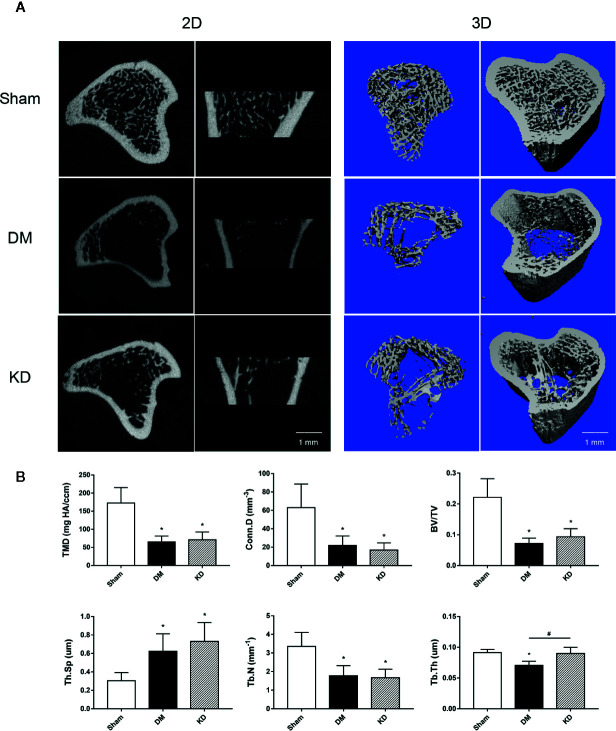
The micro-CT pictures and bone parameters of cancellous bone in the proximal tibia. **(A)** The 2D and 3D pictures of bone microstructures in the sham, diabetes mellitus (DM) and ketogenic diet (KD) groups. **(B)** The trabecular parameters of cancellous bone among groups. “*” means P < 0.05 compared to sham group, “#” means P < 0.05 between the DM and KD groups.

The micro-CT data showed that the cancellous bone was significantly compromised in the DM and KD groups compared with the sham group (**Figure 2**). The TMD and Conn.D were obviously reduced in both the DM and KD groups (the TMD and Conn.D were 172.5 mg HA/ccm and 62.9 mm-3, 65.2 mg HA/ccm and 21.8 mm-3, 71.4 mg HA/ccm and 16.8 mm-3 in the sham, DM and KD groups, respectively). And the DM and KD rats exhibited lower BV/TV and Tb.N with higher Tb.Sp than those in the sham group (the BV/TV was 22.1%, 7.2%, and 9.3% in the sham, DM and KD groups, respectively). While the Tb.Th of DM group was thinner than that of the KD group, no significant difference of Tb.Th was shown between the KD rats and the sham rats.

**Figure 3:**
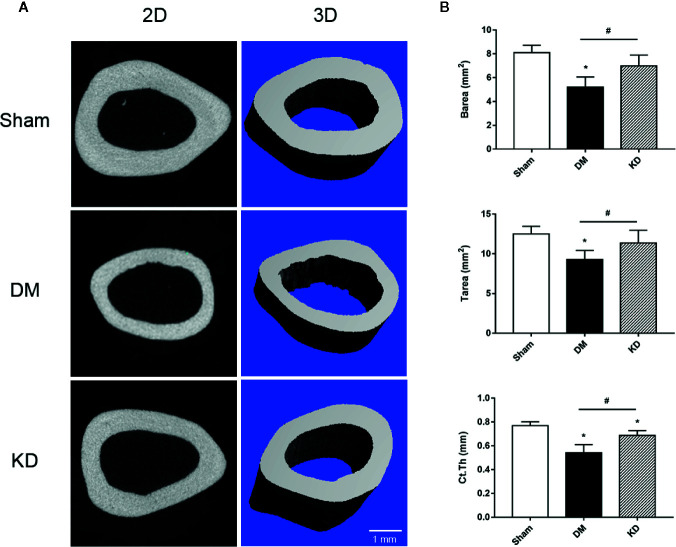
The micro-CT pictures and the bone parameters of cortical bone in the mid-shaft femur. **(A)** The 2D and 3D pictures of bone microstructures in the sham, diabetes mellitus (DM) and ketogenic diet (KD) groups. **(B)** The parameters of cortical bone in the sham, DM and KD groups. “*” means P < 0.05 compared to sham group, “#” means P < 0.05 between the DM and KD groups.

The cortical bone of the DM rats was impaired as well. The Barea and the Tarea of cortical bone were significantly decreased in the DM group compared with the sham and KD groups (**Figure 3**). The Ct.Th of the DM group was thinner than that of the sham and KD groups, although it was decreased in the KD group compared with the sham group (**Figure 3**).

### Assessments of Biomechanical Properties

**Figure 4:**
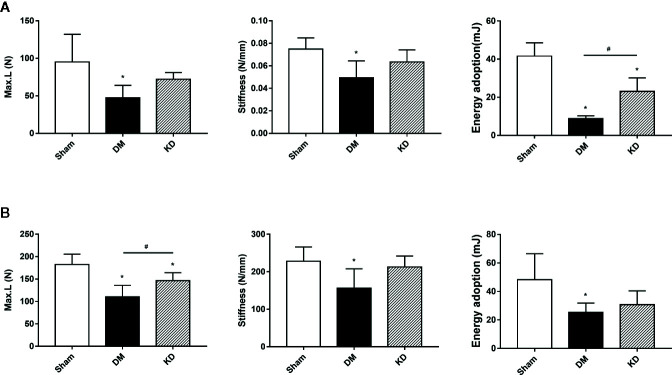
The biomechanical strengths of cortical bone in the proximal tibia **(A)** and mid-shaft femur **(B)** from the three-bending test. “*” means P < 0.05 compared to sham group, “#” means P < 0.05 between the diabetes mellitus (DM) and ketogenic diet (KD) groups.

In order to verify the biomechanical properties of the cortical bone, the three-point bending test was performed on the proximal tibia (**Figure 4A**) and the mid-shaft femur (**Figure 4B**). It was showed that the Max.L, stiffness and the energy adoption of cortical bone of tibia and femur in the DM group were significantly decreased compared with the sham group (**Figure 4**). And the Max.L and the energy adoption of tibia and femur in the KD group were decreased more noticeably than those in the sham group (**Figure 4**).

**Figure 5:**
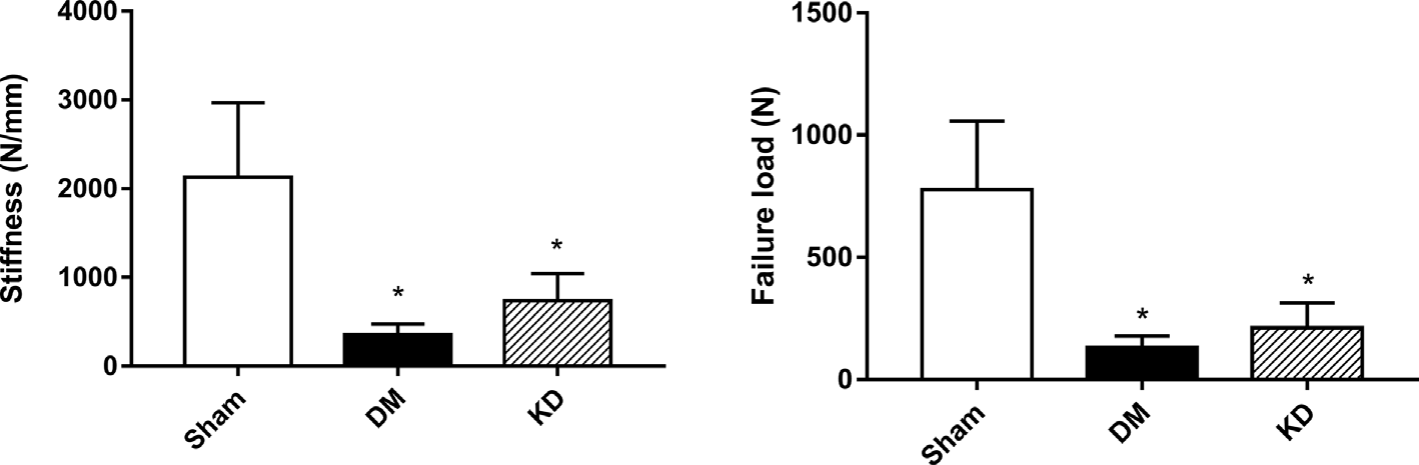
The biomechanical properties of cancellous bone in the proximal tibia from the micro-FEA. “*” means P< 0.05 compared to sham group.

The results of micro-FEA has shown that the stiffness and the failure load were significantly weakened in both the DM and KD groups compared with the sham group, and there was no significant difference between the DM and KD groups (the stiffness and failure load were 2127.3 ± 842.3 N/mm and 777.3 ± 279.6 N, 357.0 ± 117.4 N/mm and 132.1 ± 46.3 N, 734.5 ± 307.7 N/mm and 212.1 ± 100.9N in the sham, DM and KD groups, respectively) (**Figure 5**).

### Evaluations of Histology and Immunohistochemistry Staining

**Figure 6:**
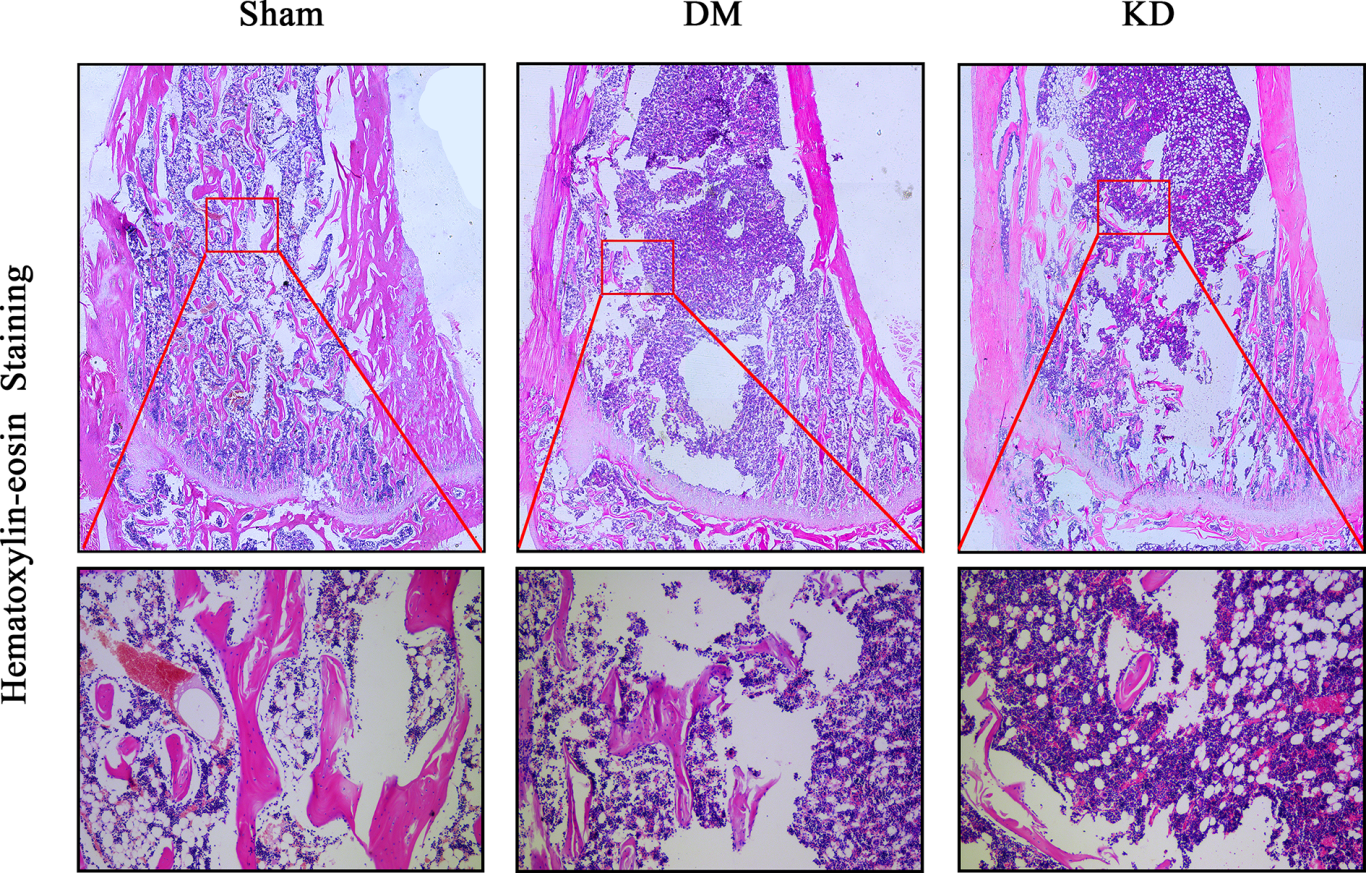
The hematoxylin-eosin stain in the proximal tibia. The trabeculae was significantly decreased in both diabetes mellitus (DM) and ketogenic diet (KD) groups compared with the sham group, and the adipose tissue (showed in red frame) in the KD group was risen compared with the sham and DM groups.

**Figure 7:**
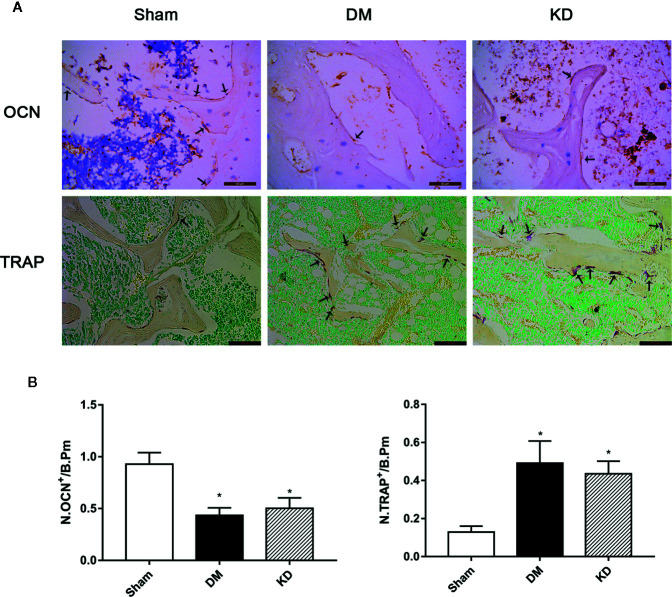
The osteocalcin (OCN) and tartrate-resistant acid phosphatase (TRAP) staining in the proximal tibia **(A)**, and the results of semi-quantitative analysis **(B)**. The activity of the osteoblast cell was significantly decreased, while the osteoclast cell was increased in diabetes mellitus (DM) and ketogenic diet (KD) groups. There was significantly lower expression of OCN and remarkably higher expression of TRAP in both DM and KD groups compared with the sham group (arrows refer to positive cells). “*” means P < 0.05 compared to sham group.

The HE staining results indicated that the number of trabeculae significantly decreased in both the DM and KD groups compared with the sham group (**Figure 6**). Meanwhile, more adipose tissue was found in the KD group than that in the sham and DM groups. Based on the immunohistochemical results, the activity of osteoblast cell was significantly decreased in the DM and KD groups, while the activity of osteoclast cell was increased. An obviously lower expression of OCN and remarkably higher expression of TRAP were identified in both the DM and KD groups relative to the sham group (**Figure 7**).

## Discussion

This study proved that both hyperglycemia and hyperketonemia induce bone compromising in rats by reducing bone mass, disturbing the balance of osteoblast and osteoclast, and impairing the biomechanical properties. Nevertheless the effects of these two kind of metabolic disturbance on bone had some differences. Hyperketonemia induced by KD resulted in more compromised more in cancellous bone with little effect on cortical bone, while hyperglycemia caused by DM had adverse effects on both the cancellous bone and cortical bone, influencing the bone length as well.

It has been long thought that diabetes mellitus negatively affects bone metabolism. Using high-resolution peripheral quantitative computed tomography and magnetic resonance imaging, several cross-sectional studies revealed that both cortical and trabecular bone tend to be more fragile in diabetes patients, which increases fracture risk (20, 21). Diabetes mellitus reduces bone density and bone strength by inhibiting osteoblast activity and enhancing osteoclast activity (22). Guo et al. (23) found that ALP activity and TRAP activity in serum were significantly decreased in the STZ-injected rats. Meanwhile, other researchers found that serum ALP level was greatly increased in diabetic rats and serum TRAP level was also greatly increased (24). The findings of TRAP activity were consistent, though the ALP level showed differences among studies. In the present study, the ALP level was remarkably reduced in the DM rats, and the TRAP concentration was significantly raised. Moreover, N-terminal propeptide of type I procollagen (P1NP) and C-telopeptide of type I collagen (CTX), as the specific biomarkers of bone formation and resorption, have been recommended for clinical application. A clinical research study into the bone turnover biomarkers showed that a corrective relationship exists between serum P1NP level and histomorphometric bone formation estimates, and between serum CTX and histomorphometric bone resorption estimates (25). The present study found that the P1NP concentration of the DM group was decreased by 56.2% and the β-CTX level increased by 143.1% in comparison with the sham group. We are inclined to the view that the functions of osteoblasts and osteoclasts were responsible for the bone loss in the DM rats which occurred at least 3 months later.

Abnormal bone metabolism indicators bring about deteriorated bone microarchitectures. Ma et al. (26) found a trabecular number and volume decrease and trabecular separation increase of femur in diabetic rats. In the present study, a remarkable TMD and Conn.D decrease was found in the diabetic rats with less bone volume fraction, lower trabecular number, thinner trabecular thickness, and wider trabecular separation. Similarly, diabetes deteriorates cortical bone and shortens bone length. Hyperglycemia increases the osteocyte lacunar density by decreasing osteocytic territorial matrix volume, thus increasing cellularity of cortical bone as well as affecting the bone length (27). The STZ-rats displayed an apparently smaller cortical area and a higher cortical porosity (28). The micro-CT data showed that the bone area, the total area and cortical thickness of the mid-shaft of femur were significantly decreased in the DM rats than those in the sham rats. The lengths of tibia and femur of the diabetic rats were remarkably shortened. The bone trabecular and cortical bone parameters exhibited that diabetes not only affects the status of cancellous bone and cortical bone, but also affects the bone growth.

The status of cortical bone plays a critical role in bone strength. Supportive findings have shown that diabetes mellitus is associated with increased risk of hip fracture and other fractures (29). In the rodent models, diabetes significantly weakened bone strengths by impairing cortical bone microarchitectures (30, 31). Three-point bending test has been proven as a useful method to measure biomechanical properties of cortical bone in rodents (32). In this study, the biomechanical properties of cortical bone of the diabetic rats were significantly weakened, which was manifested as lower maximum load, stiffness and energy absorption, compared with the sham group. Moreover, simulated compressive test based on micro-finite element analysis is an effective technique for evaluation of cancellous bone since it has an irregular structures. Consistent with the bone parameters from micro-CT, the micro-FEA results also showed that the stiffness and failure load were significantly decreased in the DM group compared with the sham group. The results from the biomechanical tests indicated that both the cortical bone and cancellous bone were deteriorated in the diabetic rats.

Researches on the effects of hyperketonemia induced by KD on bone microstructures and bone qualities in rodents were performed in our previous studies. KD deteriorated microstructures of cancellous bone by decreasing bone mass and impairing biomechanical properties, as well as inhibiting osteogenic process and enhancing osteoclastic process in the BMSCs (15, 17). Akihiro et al. (33) found that ketone bodies bidirectionally modulate osteoblast functions, which suggests that ketone bodies is an important endogenous factor that regulates bone metabolism in both physiological and pathological situations. And they pointed out that β-hydroxybutyrate significantly reduced the ALP activity and mineralization in osteoblasts. It was confirmed in this study that hyperketonemia severely impaired cancellous bone and its’ biomechanical properties, and cortical bone was partly affected. The underlying mechanism might be that hyperketonemia inhibits the osteoblast activity and increases the osteoclast activity.

Both hyperglycemia and hyperketonemia have obvious adverse effects on bone. The results of this study showed that both hyperglycemia and hyperketonemia deteriorated cancellous bone and weakened its biomechanical properties, which might be attributed to the changes of activity of osteoblasts and osteoclasts. However, there were differences existing between the two effects. Firstly, hyperglycemia had more adverse effects on cortical bone than hyperketonemia. It was found that the DM rats exhibited smaller Barea and Tarea, and thinner Ct.Th than the sham and KD rats. The Ct.Th was thinner in the KD rats compared to the sham group, which was consistent with the results of biomechanical properties. Secondly, hyperglycemia induced by diabetes significantly inhibited the bone growth. The lengths of tibia and femur in the DM rats were remarkably shorter than those in the sham and KD groups. In addition, hyperglycemia induced by diabetes and hyperketonemia induced by ketogenic diets provided similar effects on the bone turnover markers, which were decreasing the activity of osteoblasts and promoting the activity of osteoclasts. However, there was still a slight difference. According to the HE staining result, more adipocytes were found in the bone marrow cavity of the KD rats when compared with the sham and DM rats. This indicates that KD might have a stronger ability to differentiate BMSCs from osteoblasts to adipocytes.

It should be noted that the affecting mechanisms of hyperglycemia and hyperketonemia on bone are still unknown. Insulin-like growth factor 1 (IGF-1) plays a central role in growth, development and metabolism, and it contributes to normal longitudinal bone growth and cortical bone size as well as the maintenance of bone mass in adults (34). IGF-1 is highly expressed in osteoblasts and chondrocytes, and it brings its bone anabolic effects into play through promoting osteoblasts differentiation and bone mineralization (35, 36). IGF-1’s stimulation of osteoblast differentiation is a result of activation of mammalian target of rapamycin (mTOR) through the PI3K/Akt pathway (37). Ma et al. (26) indicated that diabetes may damage bone microarchitectures and bone strengths through Sema3A/IGF-1/β-catenin signaling pathway. In our previous study, KD reduced serological IGF-1 and delayed the spinal fusion (38). Bielohuby et al. (39) pointed out that diet-induced reduction in GH/IGF system components probably aggravated the bone phenotype, which might be an important factor in the KD-induced bone loss. The serological IGF-1 level decreased in both the DM and KD groups, with a lower trend in the DM group. Therefore, the systemic changes of IGF-1 might, in some extent, explain the mechanism of hyperglycemia and hyperketonemia on bone growth, bone development, and bone mass accrual. In addition, advanced glycation end products (AGEs) are diverse compounds that generated *via* a non-enzymatic reaction between reducing sugars and the amine residues on proteins, lipids, and nucleic acids. Growing evidence concerning bone fracture in patients with diabetes mellitus indicates the crucial roles of AGEs in aggravating bone fragility (40). However, ketogenic diet improves glycemic control in type 2 diabetes (41), and 3-β-hydroxybutyrate (the main component of a ketone body) inhibits the glycation process, decreases glucose binding to the protein, and prevents the formation of AGEs (42). Therefore, AGEs might not be a common role in the bone deterioration induced by hyperglycemia and hyperketonemia, but further study is needed.

Certain limitations of this study must be mentioned. On one hand, the effects of hyperglycemia and hyperketonemia on bone were observed at a single time point. The diﬀerent time points and dynamic changes of bone microstructures and histomorphometry (such as calcein stain) need to be further studied. On the other hand, the present study has only indicated that hyperglycemia and hyperketonemia have adverse effects on bone structures, but the underlying mechanisms should be further investigated.

In conclusion, the present study comprehensively compared the effects of hyperglycemia and hyperketonemia on the bone structures from various perspectives, such as serum biomarkers, bone length, biomechanical characteristics, bone microarchitectures, and the histomorphometry. The disturbance of carbohydrate metabolism and lipid metabolism have significant adverse effects on the bone tissues, which is worth calling more attention to in clinical practice.

## Data Availability Statement

All datasets generated for this study are included in the article/supplementary files.

## Ethics Statement

The animal study was reviewed and approved by Animal Ethics Committee of Southern Medical University.

## Author Contributions

QL, QZ and YuH contributed to the design of the experiment. QL and ZY performed the experiments, researched data, and wrote the manuscript. CX, LL and HH collected the data. YC and YaH provided statistical analyses. QL and YuH reviewed and edited the manuscript. All authors contributed to the article and approved the submitted version.

## Funding

This work was supported by a grant from National Natural Science Foundation of China (No. 81574002).

## Conflict of Interest

The authors declare that the research was conducted in the absence of any commercial or financial relationships that could be construed as a potential conflict of interest.
